# Etiologic classification of suspected MINOCA using cardiovascular magnetic resonance reports: a comparison of a large language model and human readers

**DOI:** 10.1007/s10554-026-03689-7

**Published:** 2026-03-23

**Authors:** Mihály Károlyi, Verena C. Wilzeck, Lucas Tramèr, Léon Groenhoff, Jochen von Spiczak, Tamar Bigvava, Hatem Alkadhi, Robert Manka

**Affiliations:** 1 Diagnostic and Interventional Radiology, University Hospital Zurich, University of Zurich, Zurich, Switzerland; 2 Department of Cardiology, University Heart Center, University Hospital Zurich, University of Zurich, Raemistrasse 100, Zurich, 8091 Switzerland; 3 Institute for Biomedical Engineering, University and ETH Zurich, Zurich, Switzerland

**Keywords:** Diagnostic classification, Large language model, Magnetic resonance imaging, MINOCA

## Abstract

The study explored the feasibility of using a large language model (LLM) for etiologic classification in patients with suspected myocardial infarction with non-obstructive coronary arteries (MINOCA) based on cardiovascular magnetic resonance (CMR) reports. We included 156 patients with MINOCA from a prospective (*n* = 50) and a retrospective (*n* = 106) pooled cohort. A large language model and three human readers with different experience levels independently classified CMR reports into eight predefined diagnostic categories, using the final expert CMR diagnosis as the reference standard. Performance and agreement were assessed using standard multiclass classification metrics. In the pooled cohort, the LLM achieved an exact-match diagnostic accuracy of 67.3% (95% CI 59.6–74.2%), lower than the expert reader (80.1%, 95% CI 73.2–85.6) but comparable to the intermediate and junior readers (both 68.6%, 95% CI 60.9–75.4%). Diagnosis-specific analysis showed consistently high specificity for the LLM (mean 94.9%), with sensitivities up to 86.0% and F1-scores up to 85.1% for common etiologies. Agreement between the LLM and the reference standard was substantial (ICC 0.84, 95% CI: 0.79–0.89) and comparable to experienced readers, whereas agreement with the junior reader was markedly lower, indicating greater diagnostic variability despite similar accuracy. A large language model shows promise as a supportive tool for etiologic classification in suspected MINOCA from CMR reports, with performance comparable to less experienced readers and high agreement with the final expert CMR diagnosis. Integration into structured reporting workflows may enhance diagnostic consistency in routine clinical practice.

**Graphical abstract:**

**Figure:**
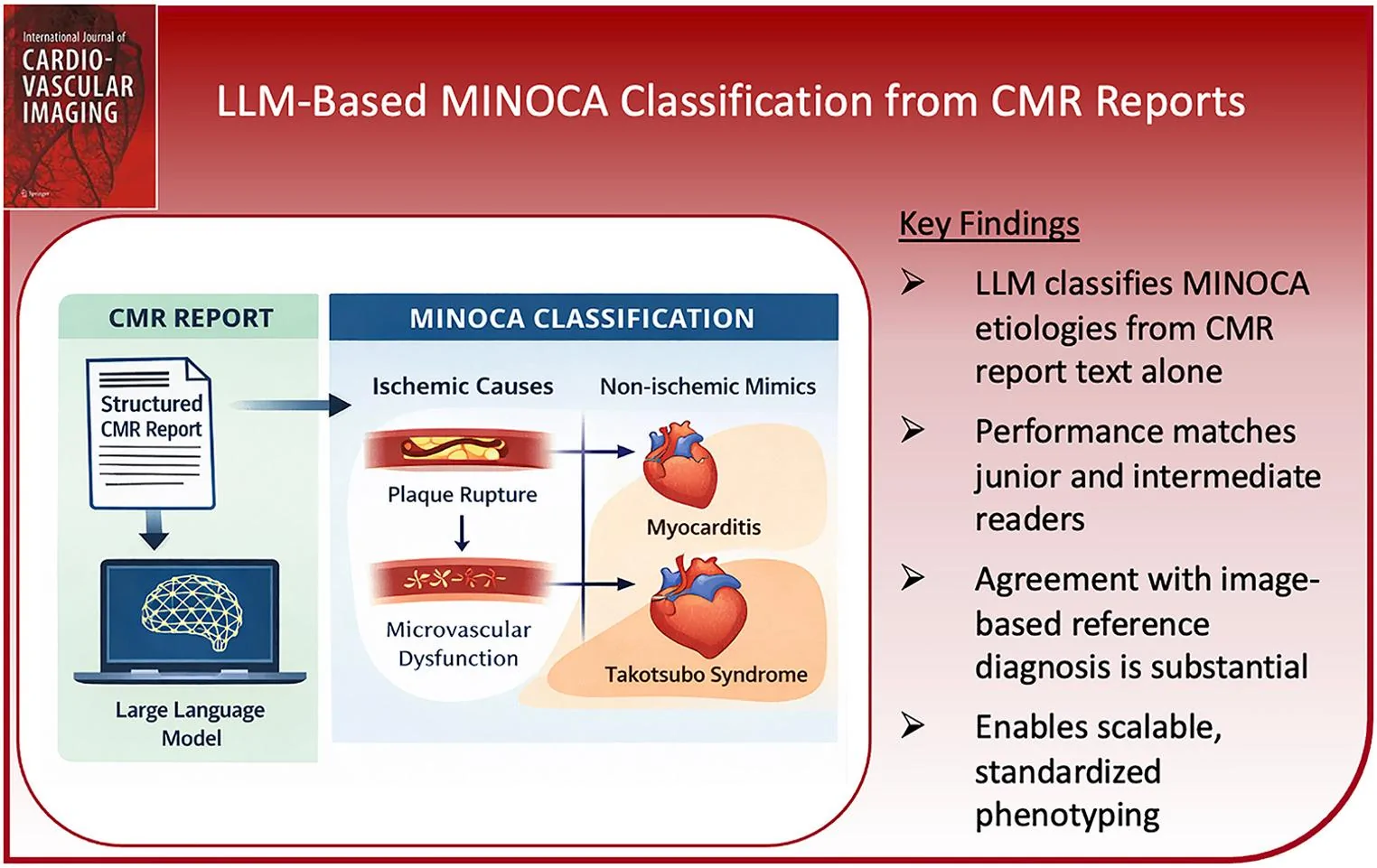

**Supplementary Information:**

The online version contains supplementary material available at https://doi.org/10.1007/s10554-026-03689-7.

## Introduction

Myocardial infarction with non-obstructive coronary arteries (MINOCA) is a working diagnosis that applies to patients fulfilling the universal criteria for acute myocardial infarction in the absence of obstructive coronary artery disease, accounting for approximately 5–10% of acute myocardial infarction presentations [1–3]. MINOCA represents a clinically heterogeneous entity and requires systematic diagnostic evaluation to identify the underlying cause. Following comprehensive assessment, a subset of patients is ultimately diagnosed with ischemic mechanisms such as coronary plaque disruption, coronary embolism or thrombosis, spontaneous coronary artery dissection, coronary vasospasm, or microvascular dysfunction [4–6]. Importantly, a substantial proportion of patients initially classified as MINOCA are subsequently found to have non-ischemic conditions, including myocarditis or Takotsubo syndrome, which are now regarded as MINOCA mimics rather than true infarction [5]. Accurate etiologic classification is essential, as prognosis, management strategies, and follow-up differ substantially between ischemic MINOCA and non-ischemic mimics [3, 6].

Cardiovascular magnetic resonance (CMR) has emerged as the cornerstone imaging modality for the diagnostic workup of MINOCA, enabling comprehensive assessment of myocardial function, tissue characterization, and scar distribution [7–9]. When interpreted by experienced readers, CMR allows etiologic classification in the majority of cases and is strongly recommended by contemporary guidelines [10, 11]. However, CMR interpretation remains complex and reader-dependent, particularly in MINOCA, where findings may be subtle, overlapping, or atypical. Interobserver variability has been reported even among trained readers, and access to highly experienced CMR expertise is not universally available in routine clinical practice [12, 13].

Recent advances in large language models (LLMs) have demonstrated their ability to extract, synthesize, and reason over complex medical text [14, 15–16]. In cardiovascular imaging, LLMs have shown promise in analyzing structured and semi-structured radiology reports, supporting diagnostic classification tasks without direct access to imaging data [17–19]. Given that CMR reports already represent a condensed expert interpretation of imaging findings, LLM-based analysis of report text may offer a scalable approach to clinical decision support, particularly in settings with limited subspecialty expertise. However, data on the diagnostic performance and reliability of LLMs in the specific context of MINOCA classification remain scarce.

The aim of this study was to explore the potential role of a large language model in the etiologic classification of MINOCA based on CMR reports. Diagnostic performance and interobserver agreement of the LLM were compared with those of human readers with different levels of experience, using the final expert CMR diagnosis as the reference standard. We sought to determine whether report-based classification using an LLM can approximate human performance in this task and how its agreement patterns compare across reader experience levels in both a controlled, research-oriented reporting environment and a heterogeneous real-world clinical setting.

## Materials and methods

### Study design and population

This study integrated data from two complementary MINOCA cohorts to evaluate report-based diagnostic classification across both protocol-driven and real-world clinical settings.

The first cohort consisted of patients prospectively enrolled in a dedicated MINOCA study (*n* = 50). Consecutive patients with a working diagnosis of MINOCA underwent standardized CMR imaging and provided written informed consent for the use of their data for research purposes. The original MINOCA study received ethical approval from the Cantonal Ethics Committee of Zurich under reference number PB_2016 − 01985 and was subsequently re-submitted and approved for extended analysis under reference number 2025 − 00466.

The second cohort comprised patients retrospectively identified through a radiology information system (RIS) search for referrals with suspected or confirmed MINOCA between September 2017 and September 2025. After application of the predefined inclusion and exclusion criteria, the final retrospective cohort consisted of 106 patients, reflecting routine clinical practice.

Together, these cohorts formed the pooled study population, enabling evaluation of report-based diagnostic classification in both a protocol-driven research setting and a real-world clinical environment.

### Inclusion and exclusion criteria

Patients were eligible for inclusion if they had undergone CMR for evaluation of suspected MINOCA and if the CMR report contained sufficient information for myocardial tissue characterization, including assessment of edema and late gadolinium enhancement. All included CMR reports were structured and followed an identical standardized reporting template across the study period. This template was aligned with internationally recommended frameworks for standardized CMR acquisition, interpretation, and reporting, ensuring consistent documentation of key diagnostic elements relevant to myocardial tissue characterization [20, 21].

For the prospective cohort, all enrolled patients fulfilled the study inclusion criteria and had provided written informed consent for research use of their data; therefore, no cases were excluded.

For the retrospective cohort, inclusion was based on the institution’s general consent framework and was restricted to patients who had not objected to the retrospective use of their clinical data for research purposes. A total of 130 patients were initially identified through the RIS search. Of these, 16 patients were excluded because they had actively declined the use of their medical data for research purposes, six cases were excluded due to incomplete tissue characterization, and two examinations were performed without contrast administration due to patient refusal, preventing full tissue characterization. No cases were excluded due to non-diagnostic image quality, as poor image quality was not documented in the clinical reports.

Patients with known or previously established obstructive coronary artery disease, cardiomyopathies, or systemic or infiltrative diseases were not included, as these conditions had been excluded during the initial clinical workup prior to referral for CMR or study enrollment [5]. All patients therefore had a working diagnosis of MINOCA at the time of CMR.

### CMR protocol and reference standard

CMR examinations were performed according to institutional MINOCA protocols. Imaging included cine sequences for ventricular volumes and function; T2-weighted imaging and native T1 and T2 mapping for myocardial tissue characterization; and late gadolinium enhancement (LGE) imaging following contrast administration [9]. Post-contrast T1 mapping was available in a subset of patients; however, extracellular volume quantification was not consistently performed and was therefore not systematically analyzed. All patients in the prospective cohort were scanned on a 1.5 Tesla Philips system. In the retrospective cohort, examinations were performed on 1.5 Tesla Philips and 1.5 or 3.0 Tesla Siemens scanners, reflecting routine clinical practice. The reference standard was defined as the final CMR diagnosis established by expert interpretation of the complete imaging dataset at the time of clinical reporting. Clinical CMR reports were generated by three experienced CMR readers (Level 3 according to EACVI criteria) as part of routine clinical practice, and these readers were not involved as observers in the present study. For the purposes of this study, the original clinical diagnoses were mapped to the eight predefined diagnostic categories used in the analysis. For the retrospectively extracted cohort, this mapping was performed by one experienced Level 3 CMR reader to ensure consistent categorization according to the study classification scheme. This final expert CMR diagnosis served as the reference standard for all analyses.

### Diagnostic categories

Each case was classified into one of eight predefined etiologic categories based on CMR findings: (1) (peri-)myocarditis, (2) (myo-)pericarditis, (3) Takotsubo syndrome, (4) acute or subacute myocardial infarction, (5) ischemic myocardial injury due to coronary embolism, thrombosis, or dissection, (6) MINOCA without an explanatory CMR finding, (7) cardiomyopathy (hypertrophic, dilated, and arrhythmogenic right or left ventricular cardiomyopathies), and (8) systemic or infiltrative disease [5, 7]. Category 5 represents cases with an ischemic myocardial injury pattern on CMR consistent with myocardial infarction occurring in the clinical context of non-obstructive coronary arteries. While CMR can identify an ischemic injury pattern based on characteristic late gadolinium enhancement distribution, it cannot determine the specific underlying coronary mechanism (e.g., embolism, thrombosis, or dissection). The category therefore reflects ischemic myocardial injury compatible with MINOCA rather than a direct CMR diagnosis of a specific coronary pathology. Category 6 refers to cases in which CMR did not reveal imaging findings that could explain the acute clinical presentation. This category therefore includes both patients with completely normal CMR and cases with incidental or chronic findings (e.g., old infarct scar or non-specific fibrosis) that were not considered related to the acute clinical episode. Coronary spasm was not defined as a separate diagnostic category, as it cannot be reliably differentiated on the basis of CMR findings or report text alone without invasive or provocative coronary angiography. These categories reflected routine clinical reporting practice and were applied consistently across all observers.

Each case was assigned a single diagnostic label corresponding to the final expert CMR diagnosis documented in the original clinical report. The study was therefore designed as a single-label multiclass classification task. In cases with overlapping imaging features, classification followed the dominant imaging pattern and the final clinical interpretation.

### Human reader assessment

Three human readers independently reviewed the written CMR reports, with access restricted to the descriptive findings section only and without access to the impression/conclusion section, imaging data, or clinical information. The descriptive findings consisted of structured narrative descriptions of imaging observations, including ventricular volumes and systolic function, regional wall motion, atrial dimensions, myocardial tissue characterization (including T1 and T2 mapping values where available), and the presence, localization, and pattern of late gadolinium enhancement. Readers were categorized according to experience level based on European Association of Cardiovascular Imaging (EACVI) criteria: Reader 1 (R1) expert (Level 3); Reader 2 (R2) intermediate (Level 2); and Reader 3 (R3) junior (Level 1) [22]. All readers were blinded to the reference diagnosis and to each other’s assessments and assigned a single etiologic category per case.

### Large language model assessment

A single large language model (ChatGPT, OpenAI, GPT-4 architecture) was used for report-based classification [23]. For each case, the anonymized CMR report text was provided to the model, with input restricted to the descriptive findings section and without access to the original impression/conclusion, imaging data, clinical follow-up, or information from other cases. The model was instructed to assign one of eight predefined diagnostic categories using an independent prompt for each case. A fixed instruction prompt was used for all cases. The prompt asked the model to read the CMR report (written in German) and assign the case to one of the predefined diagnostic categories based solely on the information contained in the report text. No prompt optimization or systematic testing of alternative prompt formulations was performed; the prompt was intentionally designed as a simple baseline instruction rather than task-specific prompt engineering. To minimize contextual bias, a newly created ChatGPT account without prior interaction history was used, and each case was evaluated in a separate, independent conversation session. Model performance was assessed based on the first response generated for each case following submission of the prompt and the anonymized CMR report text. No repeated runs or averaging across multiple outputs were performed. This approach was chosen to reflect a realistic clinical workflow in which an LLM would be queried once per case. The full prompt used for the evaluation is provided in Supplementary Material 1 to ensure transparency and reproducibility.

### Assessment of reporting time

Reporting time was measured for each case for LLM-based classification and for three human readers with different experience levels. Time was defined as the duration required to assign a final CMR-based diagnostic category and was recorded in seconds for all assessments. Reporting times were compared descriptively between LLM and human readers.

### Statistical analysis

Continuous variables are reported as median with interquartile range, and categorical variables as counts and percentages. Diagnostic performance was primarily assessed using exact-match multiclass accuracy, defined as complete agreement between the assigned diagnosis and the reference standard. Diagnosis-specific performance was additionally evaluated using a one-vs-rest approach, reporting sensitivity, specificity, and F1-score for each category. Exact binomial 95% confidence intervals were calculated for accuracy estimates. Interobserver agreement between observers and the reference standard, as well as between human readers, was quantified using intraclass correlation coefficients (ICC; two-way random-effects model, absolute agreement) [24]. Overall agreement among the three human readers was assessed using Fleiss’ kappa [25]. A two-sided p-value < 0.05 was considered statistically significant. Statistical analyses were performed using SPSS (ver. 30; Armonk, NY).

## Results

### Participants

A total of 156 patients with MINOCA were included, comprising a prospectively enrolled cohort of 50 patients (42 men; median age 52.1 years, IQR 41.0–59.0) and a retrospective RIS-derived cohort of 106 patients (56 men; median age 57.6 years, IQR 46.5–70.3). Final CMR-based diagnoses are summarized in Table 1.

**Table 1:** Distribution of final expert CMR diagnosis in the two patient cohorts

Consensus CMR diagnosis	Cohort 1 (*n* = 50)	Cohort 2 (*n* = 106)
(Peri-)Myocarditis, n (%)	20 (40)	21 (19.8)
(Myo-)Pericarditis, n (%)	1 (2)	4 (3.8)
Takotsubo syndrome, n (%)	2 (4)	7 (6.6)
True acute or subacute myocardial infarction, n (%)	11 (22)	16 (15.1)
Ischemic Injury (coronary embolism, thrombosis or dissection), n (%)	4 (8)	22 (20.8)
CMR without explanatory finding, n (%)	12 (24)	31 (29.2)
Cardiomyopathy, n (%)	0 (0)	4 (3.8)
Systemic or infiltrative disease, n (%)	0 (0)	1 (0.9)

Values are presented as number of patients (percentage within cohort). Diagnostic categories were assigned using a predefined CMR-based classification scheme applied uniformly across both cohorts

### Overall diagnostic accuracy

**Fig. 1:**
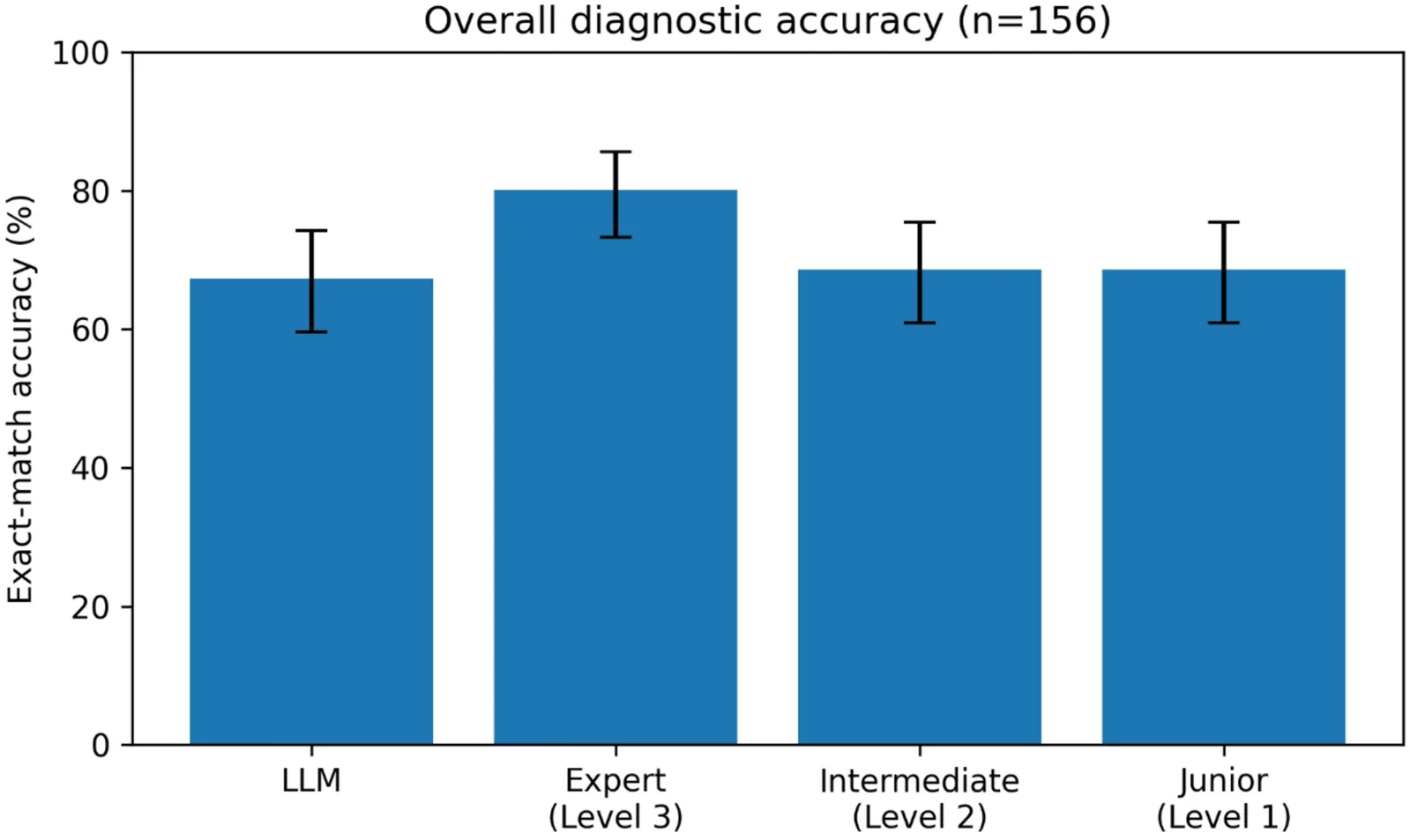
Overall diagnostic accuracy. Overall exact-match multiclass diagnostic accuracy with 95% confidence intervals for the large language model (LLM) and three human readers stratified by experience level (expert, intermediate, junior) in the pooled MINOCA cohort (*n* = 156). The expert reader corresponds to European Association of Cardiovascular Imaging Level 3 training, the intermediate reader to Level 2, and the junior reader to Level 1

Overall diagnostic performance was primarily assessed using exact-match multiclass accuracy. In the pooled cohort, the LLM achieved an exact-match accuracy of 67.3% (95% CI: 59.6–74.2). The expert reader (Level 3) demonstrated higher performance with an accuracy of 80.1% (95%: CI 73.2–85.6), whereas the intermediate (Level 2) and junior (Level 1) readers showed identical accuracies of 68.6% (95% CI: 60.9–75.4). Thus, exact-match performance of the LLM was lower than that of the expert reader but comparable to that of less experienced readers (Fig. 1).

When stratified by cohort, mean macro-averaged one-vs-rest accuracy of the LLM was higher in the prospectively enrolled cohort than in the retrospective cohort (94.1% vs. 90.9%), reflecting increased heterogeneity of referral indications and report structure in the real-world setting. Despite this, specificity remained high across cohorts.

Macro-averaged one-vs-rest accuracy values were substantially higher than exact-match multiclass accuracy for all observers, reflecting the predominance of true-negative classifications in low-prevalence diagnostic categories (Table 2).

**Table 2:** Overall diagnostic performance

	Cohort 1 (*n* = 50)	Cohort 2 (*n* = 106)	Pooled cohort (*n* = 156)
LLM	94.1%	90.9%	91.9%
Reader 1	95.3%	95.1%	95.2%
Reader 2	81.6%	82.0%	82.0%
Reader 3	90.7%	92.9%	92.2%

Values represent macro-averaged one-vs-rest accuracy across diagnostic categories and are therefore reported as percentages rather than numerator/denominator fractions. Confidence intervals were not calculated for macro-averaged metrics. Exact-match multiclass accuracy with 95% confidence intervals is reported separately

### Diagnosis specific performance

Diagnosis-specific performance was evaluated using a one-vs-rest approach. In the pooled analysis, the LLM showed consistently high specificity across diagnostic categories (mean specificity 94.9%), while sensitivity varied by etiology. For common diagnostic categories, sensitivity ranged from 85.2% to 86.0%, with corresponding F1-scores up to 85.1%. In contrast, performance for rare etiologies was limited by low prevalence, resulting in reduced sensitivity and non-calculable precision or F1-scores in selected categories.

**Fig. 2:**
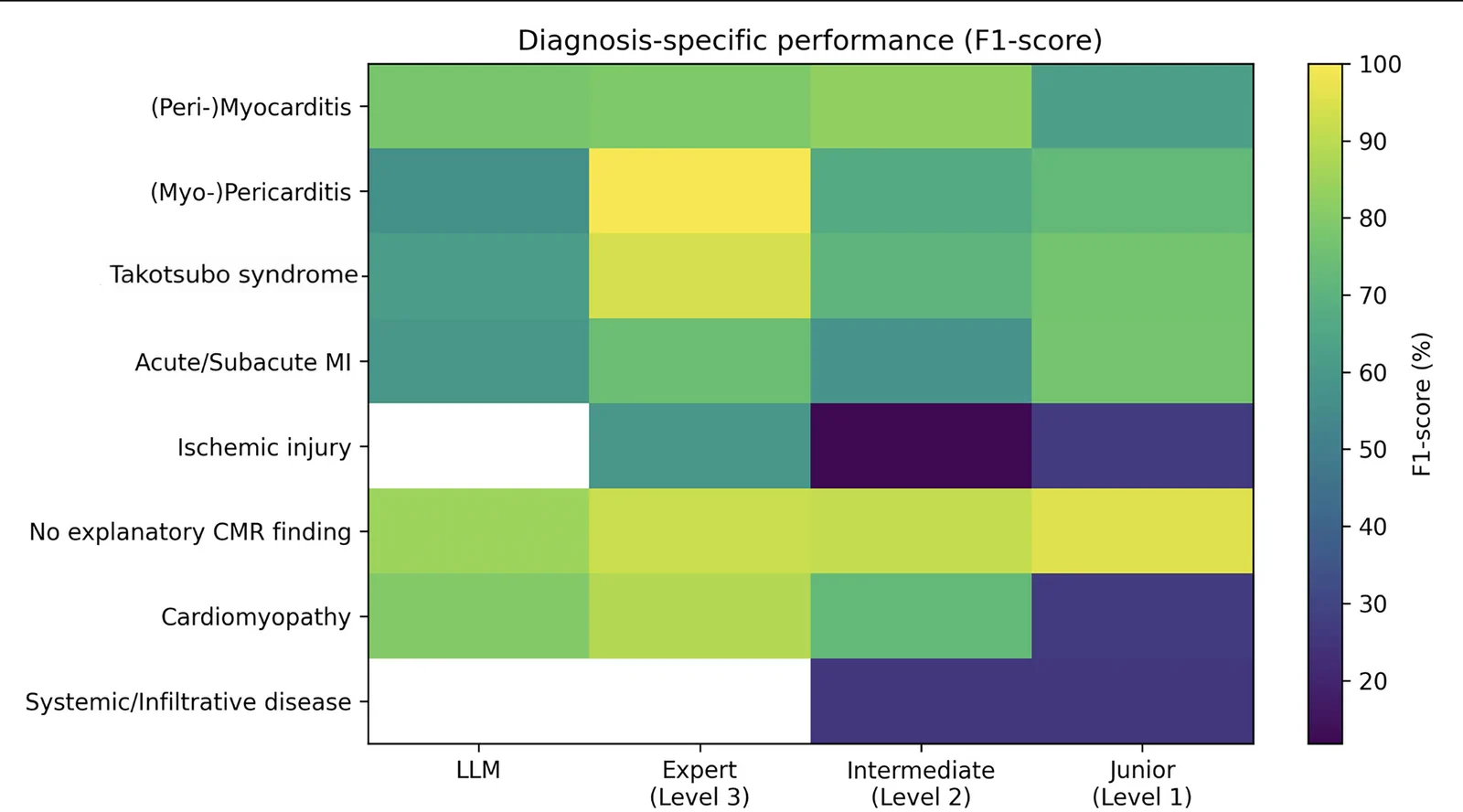
Diagnosis-specific performance across observers. Heatmap showing diagnosis-specific F1-scores (%) for the LLM and human readers using a one-vs-rest classification approach across predefined CMR diagnostic categories. Cells left blank indicate metrics that could not be calculated due to absence of positive cases in the reference standard or predicted classifications

Across all observers, diagnosis-specific performance demonstrated a clear dependence on reader experience. The expert reader consistently achieved higher accuracy and F1-scores than the intermediate and junior readers. The LLM frequently matched or exceeded the performance of less experienced readers, particularly in the retrospective cohort. Diagnosis-specific performance patterns are summarized in Table 3 and visualized in Fig. 2.

**Table 3:** Diagnosis-specific performance using a one-vs-rest approach

Diagnosis	Observer	Sensitivity	Specificity	F1-score
(Peri-)Myocarditis	LLM	85.4%	87.9%	77.8%
	R1	82.9%	90.5%	79.1%
	R2	82.9%	94.0%	82.9%
	R3	47.6%	98.3%	62.5%
(Myo-)Pericarditis	LLM	40.0%	100%	57.1%
	R1	100%	100%	100%
	R2	60.0%	99.3%	66.7%
	R3	100%	98.0%	72.7%
Takotsubo syndrome	LLM	44.4%	100%	61.5%
	R1	88.9%	100%	94.1%
	R2	66.7%	98.6%	70.6%
	R3	62.5%	100%	76.9%
Acute or subacute myocardial infarction	LLM	85.2%	79.2%	59.7%
	R1	81.5%	92.3%	74.6%
	R2	77.8%	81.5%	58.3%
	R3	79.1%	90.4%	77.3%
Ischemic myocardial injury	LLM	–	99.2%	-
(embolism, thrombosis or dissection	R1	46.2%	98.5%	60.0%
pattern)	R2	77.0%	95.4%	11.8%
	R3	22.2%	97.3%	26.7%
MINOCA without explanatory CMR	LLM	86.0%	94.2%	85.1%
finding	R1	95.3%	95.8%	92.1%
	R2	83.7%	100%	91.1%
	R3	94.7%	99.2%	96.0%
Cardiomyopathy	LLM	100%	98.7%	80.0%
	R1	100%	99.3%	88.9%
	R2	100%	98.0%	72.7%
	R3	57.1%	87.3%	26.7%
Systemic or infiltrative disease	LLM	–	100%	–
	R1	–	98.7%	–
	R2	100%	–	25.0%
	R3	40.0%	94.1%	25.0%

Diagnosis-specific sensitivity, specificity, and F1-score were calculated using a one-vs-rest approach for each predefined CMR diagnostic category. All values are reported as percentages. Cells marked with “–” indicate metrics that could not be calculated due to absence of positive cases in the reference standard or predicted classifications

### Interobserver reliability

Interobserver agreement demonstrated substantial agreement between the LLM and the reference standard. In the pooled cohort (*n* = 156), ICC between the reference diagnosis and the LLM was 0.84 (95% CI: 0.79–0.89), comparable to the expert and intermediate readers (expert: 0.82 [95% CI: 0.76–0.87]; intermediate: 0.82 [95% CI: 0.76–0.87]). Agreement with the junior reader was markedly lower (0.52 [95% CI: 0.33–0.65]).

**Fig. 3:**
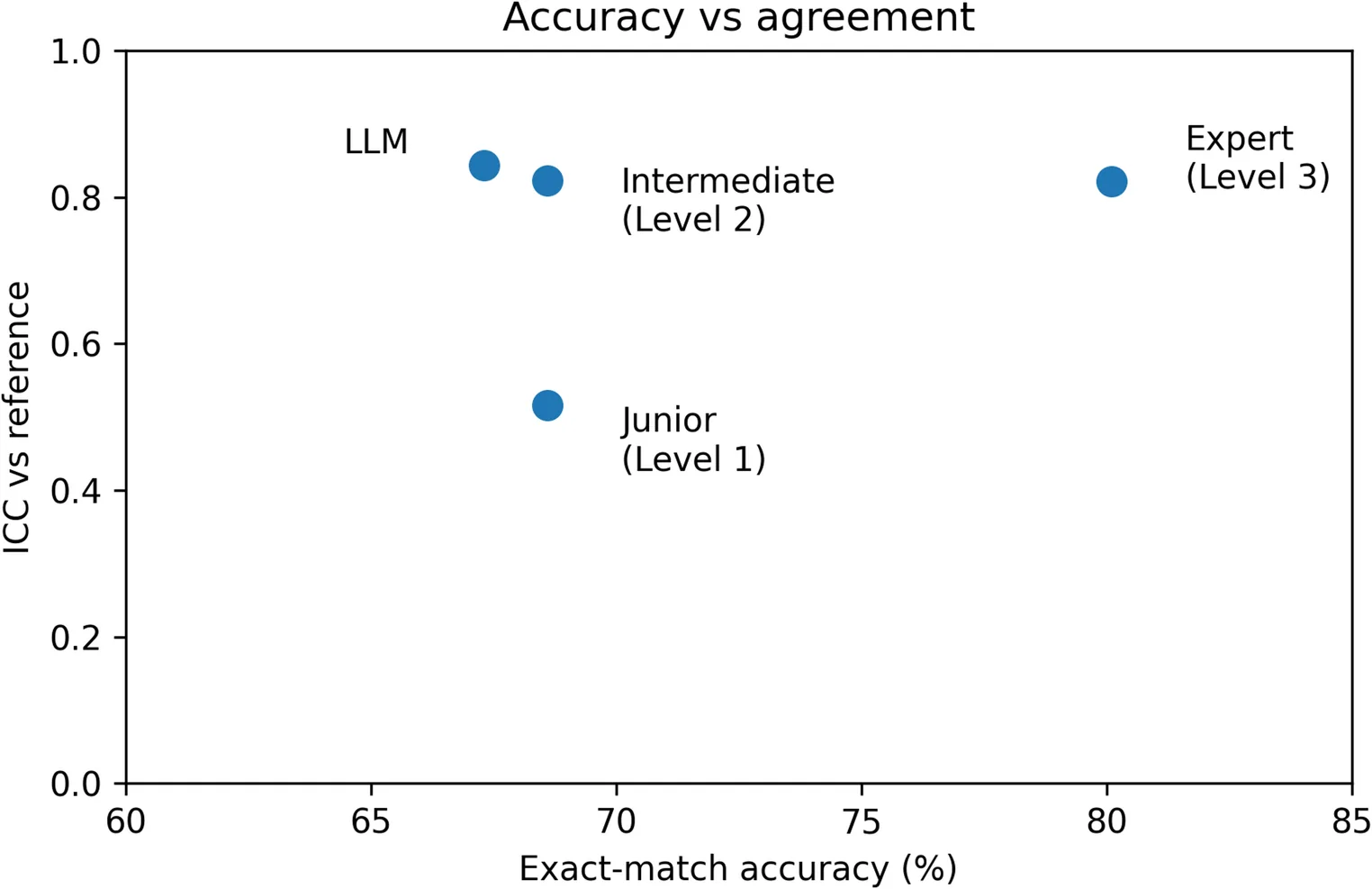
Relationship between diagnostic accuracy and interobserver agreement. Scatter plot illustrating the relationship between exact-match multiclass diagnostic accuracy (%) and interobserver agreement with the reference standard, quantified by intraclass correlation coefficients (ICC). Although intermediate and junior readers demonstrated similar accuracy, agreement with the reference standard differed substantially, highlighting that comparable accuracy does not necessarily imply comparable diagnostic consistency

Human–human agreement further demonstrated a clear experience gradient, with high agreement between the expert and intermediate readers (ICC 0.81 [95% CI: 0.73–0.86]) and substantially lower agreement involving the junior reader. Notably, although the intermediate and junior readers achieved similar exact-match diagnostic accuracy, substantially lower ICC values for the junior reader indicate greater variability and larger categorical deviations from the reference diagnosis, highlighting that comparable accuracy does not necessarily imply comparable diagnostic consistency (Fig. 3).

When stratified by cohort, ICC between the LLM and the reference standard remained high in both the prospective cohort (0.86 [95% CI: 0.76–0.92]) and the retrospective cohort (0.82 [95% CI: 0.73–0.88]), indicating stable agreement across different clinical settings. Interobserver agreement metrics are summarized in Table 4. Overall agreement among the three human readers was substantial, as reflected by Fleiss’ kappa values reported in the table legend.

**Table 4:** Interobserver agreement between the LLM, human readers, and the reference standard

	Cohort 1 (*n* = 50)	Cohort 2 (*n* = 106)	Pooled cohort	
	ICC (95% CI)	ICC (95% CI)	ICC (95% CI)	*p*-value all
LLM vs. Reference	0.86 (0.76–0.92)	0.82 (0.73–0.88)	0.84 (0.79–0.89)	< 0.0001
R1 vs. Reference	0.87 (0.77–0.93)	0.78 (0.67–0.85)	0.82 (0.76–0.87)	< 0.0001
R2 vs. Reference	0.82 (0.68–0.90)	0.82 (0.73–0.88)	0.82 (0.76–0.87)	< 0.0001
R3 vs. Reference	0.55 (0.21–0.75)	0.47 (0.23–0.64)	0.52 (0.33–0.65)	< 0.0001
R1 vs. R2	0.85 (0.73–0.91)	0.77 (0.66–0.84)	0.81 (0.73–0.86)	< 0.0001
R1 vs. R3	0.42 (0.02–0.67)	0.41 (0.15–0.60)	0.44 (0.23–0.59)	< 0.0001
R2 vs. R3	0.45 (0.07–0.68)	0.57 (0.37–0.71)	0.54 (0.36–0.67)	< 0.0001

Interobserver agreement was assessed using intraclass correlation coefficients (ICC) with 95% confidence intervals for pairwise comparisons between the LLM, human readers, and the reference standard. ICC values are reported separately for the prospectively enrolled cohort, the retrospective cohort, and the pooled analysis

Overall agreement among the three human readers was additionally assessed using Fleiss’ kappa, yielding values of 0.63 for the prospective cohort, 0.61 for the retrospective cohort, and 0.62 for the pooled cohort, indicating substantial inter-reader agreement

### Reporting time analysis

**Fig. 4:**
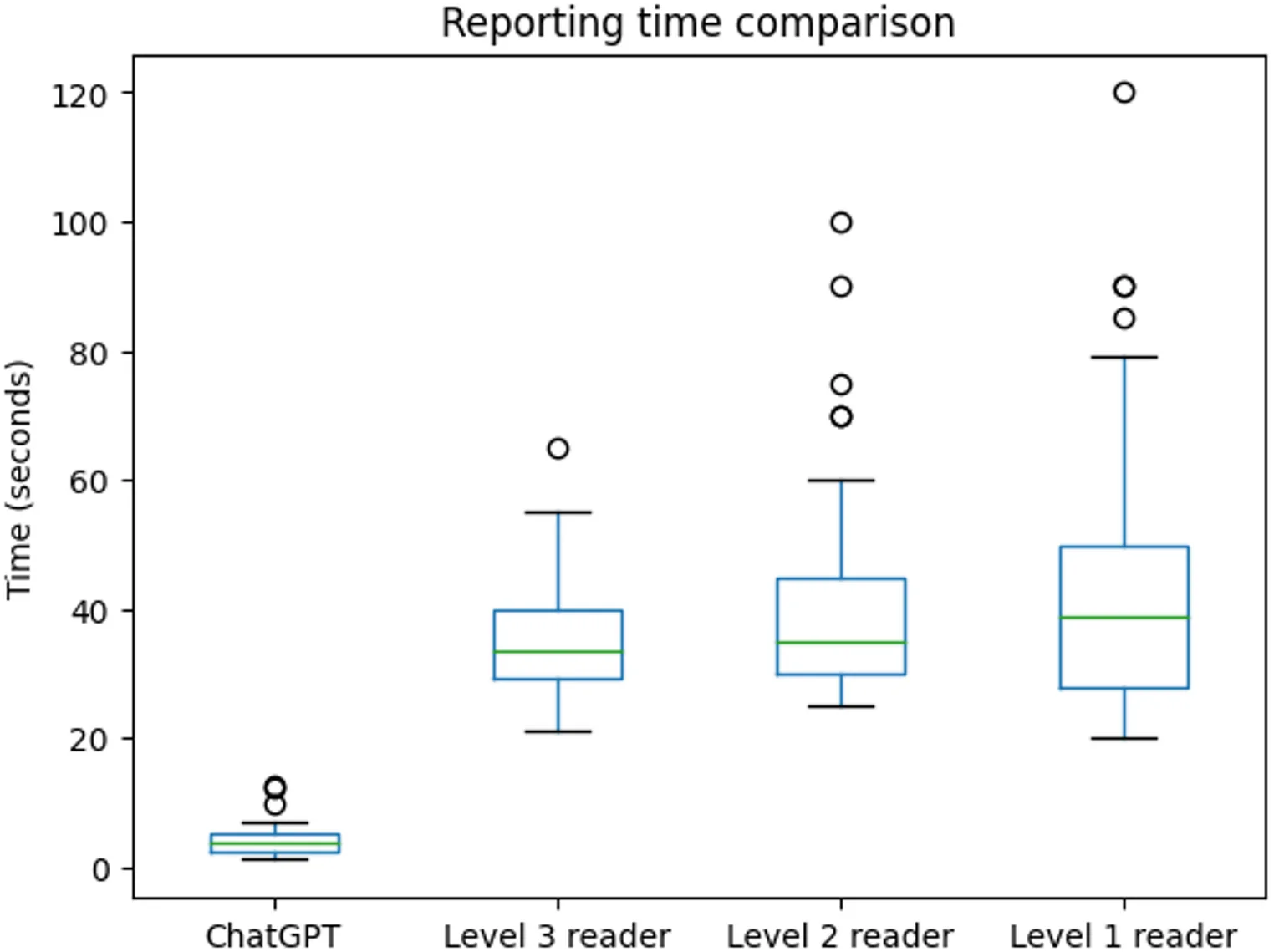
Comparison of reporting time between LLM and human readers. Boxplots illustrate the distribution of reporting times in seconds LLM and three human readers. The central line indicates the median, boxes represent the interquartile range, and whiskers indicate the range excluding outliers

Median reporting time was substantially shorter for the LLM compared with all human readers (Fig. 4). The LLM required a median of 3.8 s per case, whereas median reporting times were 33.5 s for the Level 3 reader, 35.0 s for Level 2 2, and 39.0 s for Level 1.

## Discussion

In this study, we demonstrate that a large language model can classify patients with MINOCA into etiologic subgroups using CMR report text with diagnostic performance comparable to less experienced human readers and substantial agreement with the expert reference diagnosis. This finding is clinically relevant in the context of MINOCA, a heterogeneous syndrome in which accurate phenotyping relies on nuanced interpretation of tissue characterization findings and their concordance with the index clinical presentation. Our results suggest that rather than replacing expert interpretation, report-based LLM classification may function as a scalable decision-support tool by providing pre-classification, standardized etiologic mapping, and quality-control support, such as the identification of potentially discordant or atypical cases, in routine and research settings. Such functionality may reduce cognitive workload for expert readers, facilitate harmonized phenotyping across centers, and support structured training and benchmarking of junior and intermediate readers.

Using exact-match multiclass accuracy as the primary endpoint, the LLM performed below the expert reader but similarly to intermediate and junior readers. Diagnosis-specific one-vs-rest analysis further demonstrated consistently high specificity for the LLM, with sensitivity varying across etiologic categories. Performance was strongest for common diagnostic entities and declined for rarer categories, where low prevalence limited the interpretability of precision-based metrics. This prevalence dependency is not unique to LLM-based approaches and similarly affects human readers in multiclass diagnostic settings, underscoring the importance of reporting both overall and diagnosis-specific performance measures. Although intermediate and junior readers achieved similar overall accuracy, interobserver agreement differed substantially, reflecting greater diagnostic variability and larger categorical deviations from the reference diagnosis among less experienced readers, effects that are not captured by accuracy alone.

Lower performance of the LLM in diagnosing entities such as (myo-)pericarditis and Takotsubo syndrome may reflect the dependence of report-based classification on the explicit wording of diagnostic features within the report text. In contrast to myocardial infarction or myocarditis, which often present with well-defined tissue characterization patterns, these conditions may rely on a combination of subtler imaging findings and contextual interpretation. Consequently, variability in how such findings are described in narrative reports may limit reliable extraction by a language model.

Notably, we also observed individual cases in which the LLM correctly classified reports that were misclassified by the junior reader while experienced readers reached the correct diagnosis, suggesting that LLM-based report analysis may capture explicit diagnostic cues that are also recognized by more experienced readers. This observation may highlight a potential educational role of such systems as supportive tools for less experienced readers.

Our findings align with and extend recent literature on report-based LLM application in cardiovascular imaging. Kaya et al. evaluated GPT-4 for myocarditis diagnosis based on CMR reports and clinical data in a large multicenter cohort, while explicitly withholding the final impression from the input. GPT-4 achieved an accuracy of 83% comparable to a junior reader but inferior to more experienced readers [26]. While that study addressed a binary diagnostic task in a single disease entity, our work expands the report-based LLM paradigm to multiclass etiologic classification in MINOCA, a substantially more heterogeneous diagnostic setting. The lower exact-match accuracy in our study is therefore expected, given the increased task complexity and broader differential diagnosis. Importantly, Kaya et al. also reported improved LLM performance when detailed tissue characterization information, including mapping data, was present in the report text, highlighting that report-based decision-making is sensitive to the completeness and standardization of diagnostic descriptors [26].

In the present study, all CMR reports followed a uniform, structured reporting template that was consistent across the entire study period and aligned with internationally recommended CMR reporting frameworks [20, 21]. This standardized structure likely facilitated both human and LLM-based extraction of diagnostically relevant features and reduced variability related to report heterogeneity. Accordingly, the performance observed in our study may represent an upper-bound estimate for report-based classification in environments with consistent structured reporting. At the same time, this finding underscores the importance of structured reporting for both reproducible human interpretation and robust secondary use of narrative CMR reports by automated methods.

Beyond direct diagnostic classification, several studies suggest that LLMs can contribute more broadly to radiology workflows by improving report structure, consistency, and downstream usability. Adams et al. demonstrated the feasibility of transforming free-text radiology reports into structured formats using GPT-4 [17], while Hasani et al. showed that LLMs can support report standardization across radiologic domains [27]. Salam et al. further reported that LLMs can translate CMR report content into more patient-friendly language [28]. Together, these findings suggest a coherent trajectory in which LLMs function not only as classifiers, but also as enablers of standardized reporting, reduced interobserver variability, and scalable secondary use cases such as cohort identification, registry construction, automated triage, and quality monitoring. In the context of MINOCA, where etiologic interpretation is particularly sensitive to variability in report language across centers and scanners, structured reporting may improve both human reproducibility and LLM robustness.

Notably, LLM-based classification required only a few seconds per case, resulting in an order-of-magnitude reduction in interpretation time compared with human readers. This efficiency advantage may be particularly relevant for large-scale phenotyping, retrospective analyses, and research applications where rapid and reproducible classification is required.

Several limitations should be acknowledged. First, both the LLM and human readers in this study evaluated CMR report text rather than imaging data, inherently constraining diagnostic performance compared with direct image interpretation. The output of the model cannot exceed the informational content and clarity of the report, and errors, omissions, or ambiguous phrasing in the source text may propagate to the classifier. In addition, the present study evaluated the standalone diagnostic performance of a large language model rather than an LLM-assisted reading scenario. Consequently, we did not assess whether access to LLM-generated classifications could improve diagnostic performance of human readers, particularly less experienced observers. Future studies should investigate human–AI collaborative workflows, for example by comparing reader performance with and without LLM assistance. The reporting time analysis primarily reflects the computational efficiency of automated report-based classification rather than a direct replacement of human interpretation time in routine clinical workflows. The potential value of such tools may therefore lie less in per-case time savings and more in scalable applications such as automated phenotyping, cohort identification, registry construction, and quality monitoring. Second, we evaluated a single LLM and a single prompting strategy; performance may differ across model versions, prompting approaches, or institution-specific reporting conventions. The prompt was intentionally kept fixed and non-optimized to ensure consistency across cases rather than task-specific prompt engineering. Third, rare diagnostic categories limited diagnosis-specific precision and F1-score estimation, resulting in non-calculable metrics for some observer–category combinations. This reflects data scarcity rather than intrinsic model failure and highlights the need for larger cohorts or targeted enrichment strategies when evaluating rare etiologies. Finally, although part of the dataset was derived from routine clinical practice, all included CMR reports followed a standardized structured reporting format. While this enhances internal consistency and interpretability, it may limit the linguistic richness and contextual variability available to the model and thus the generalizability of our findings to clinical settings where reports are unstructured or narrative. LLMs are inherently designed to process free-text narratives, and their performance in prose-based reporting environments remains to be determined. Future studies should therefore assess report-based LLM classification across more heterogeneous reporting formats and institutions, including direct comparisons between structured and unstructured reports.

## Conclusion

In conclusion, a general-purpose LLM without task-specific fine-tuning demonstrated report-based etiologic classification performance in MINOCA comparable to junior and intermediate readers, with substantial agreement with the final expert CMR diagnosis. These findings support the role of LLMs as decision-support tools for standardized phenotyping from routine CMR reports and establish a baseline against which future work can assess whether task-specific training, structured reporting, or hybrid human–LLM workflows may further improve robustness, reproducibility, and portability across clinical settings.

## Supplementary Information

Below is the link to the electronic supplementary material.

Supplementary Material 1

## Data Availability

The datasets used and/or analyzed during the current study are available from the corresponding author on reasonable request.

## Abbreviations

CMR: Cardiac magnetic resonance
EACVI: European Association of cardiovascular imaging
ICC: Intraclass correlation coefficient
IQR: Interquartile range
LLM: Large language model
MINOCA: Myocardial infarction with non–obstructive coronary arteries
RIS: Radiology Information System

## References

[1] Lindahl B, Baron T, Albertucci M, Prati F (2021) Myocardial infarction with non-obstructive coronary artery disease. EuroIntervention 17:e875–e887. 10.4244/EIJ-D-21-00426 PMC9724940 34870600

[2] Pasupathy S, Air T, Dreyer RP et al (2015) Systematic review of patients presenting with suspected myocardial infarction and nonobstructive coronary arteries. Circulation 131:861–870. 10.1161/CIRCULATIONAHA.114.011201 25587100

[3] Parwani P, Kang N, Safaeipour M et al (2023) Contemporary Diagnosis and Management of Patients with MINOCA. Curr Cardiol Rep 25:561–570. 10.1007/s11886-023-01874-x PMC10188585 37067753

[4] Mohammed A-Q, Abdu FA, Liu L et al (2023) Coronary microvascular dysfunction and myocardial infarction with non-obstructive coronary arteries: Where do we stand? Eur J Intern Med 117:8–20. 10.1016/j.ejim.2023.07.016 37482469

[5] Tamis-Holland JE, Jneid H, Reynolds HR et al (2019) Contemporary Diagnosis and Management of Patients With Myocardial Infarction in the Absence of Obstructive Coronary Artery Disease: A Scientific Statement From the American Heart Association. Circulation 139:e891–e908. 10.1161/CIR.0000000000000670 30913893

[6] Yildiz M, Ashokprabhu N, Shewale A et al (2022) Myocardial infarction with non-obstructive coronary arteries (MINOCA). Front Cardiovasc Med 9:1032436. 10.3389/fcvm.2022.1032436 PMC9705379 36457805

[7] Dastidar AG, Baritussio A, De Garate E et al (2019) Prognostic Role of CMR and Conventional Risk Factors in Myocardial Infarction With Nonobstructed Coronary Arteries. JACC Cardiovasc Imaging 12:1973–1982. 10.1016/j.jcmg.2018.12.023 30772224

[8] Friedrich MG, Sechtem U, Schulz-Menger J et al (2009) Cardiovascular magnetic resonance in myocarditis: A JACC White Paper. J Am Coll Cardiol 53:1475–1487. 10.1016/j.jacc.2009.02.007 PMC2743893 19389557

[9] Ferreira VM, Schulz-Menger J, Holmvang G et al (2018) Cardiovascular Magnetic Resonance in Nonischemic Myocardial Inflammation: Expert Recommendations. J Am Coll Cardiol 72:3158–3176. 10.1016/j.jacc.2018.09.072 30545455

[10] Rao SV, O’Donoghue ML, Ruel M et al (2025) 2025 ACC/AHA/ACEP/NAEMSP/SCAI Guideline for the Management of Patients With Acute Coronary Syndromes: A Report of the American College of Cardiology/American Heart Association Joint Committee on Clinical Practice Guidelines. Circulation 151:e771–e862. 10.1161/CIR.0000000000001309 40014670

[11] Byrne RA, Rossello X, Coughlan JJ et al (2023) 2023 ESC Guidelines for the management of acute coronary syndromes. Eur Heart J 44(38):3720–3826. 10.1093/eurheartj/ehad191 37622654

[12] Károlyi M, Polacin M, Kolossváry M et al (2024) Comparative analysis of late gadolinium enhancement assessment techniques for monitoring fibrotic changes in myocarditis follow-up. Eur Radiol 34:7264–7274. 10.1007/s00330-024-10756-x PMC11519139 38703188

[13] Kotanidis CP, Bazmpani M-A, Haidich A-B et al (2018) Diagnostic Accuracy of Cardiovascular Magnetic Resonance in Acute Myocarditis: A Systematic Review and Meta-Analysis. JACC Cardiovasc Imaging 11:1583–1590. 10.1016/j.jcmg.2017.12.008 29454761

[14] Floridi L, Chiriatti M (2020) GPT-3: Its Nature, Scope, Limits, and Consequences. Minds Machines 30:681–694. 10.1007/s11023-020-09548-1

[15] Clusmann J, Kolbinger FR, Muti HS et al (2023) The future landscape of large language models in medicine. Commun Med (Lond) 3:141. 10.1038/s43856-023-00370-1 PMC10564921 37816837

[16] Liu M, Okuhara T, Chang X (2024) Performance of ChatGPT Across Different Versions in Medical Licensing Examinations Worldwide: Systematic Review and Meta-Analysis. J Med Internet Res 26:e60807. 10.2196/60807 PMC11310649 39052324

[17] Adams LC, Truhn D, Busch F et al (2023) Leveraging GPT-4 for Post Hoc Transformation of Free-text Radiology Reports into Structured Reporting: A Multilingual Feasibility Study. Radiology 307:e230725. 10.1148/radiol.230725 37014240

[18] Kottlors J, Bratke G, Rauen P et al (2023) Feasibility of Differential Diagnosis Based on Imaging Patterns Using a Large Language Model. Radiology 308:e231167. 10.1148/radiol.231167 37404149

[19] Gertz RJ, Bunck AC, Lennartz S et al (2023) GPT-4 for Automated Determination of Radiological Study and Protocol Based on Radiology Request Forms: A Feasibility Study. Radiology 307:e230877. 10.1148/radiol.230877 37310247

[20] Kramer CM, Barkhausen J, Bucciarelli-Ducci C et al (2020) Standardized cardiovascular magnetic resonance imaging (CMR) protocols: 2020 update. J Cardiovasc Magn Reson 22:17. 10.1186/s12968-020-00607-1 PMC7038611 32089132

[21] Schulz-Menger J, Bluemke DA, Bremerich J et al (2020) Standardized image interpretation and post-processing in cardiovascular magnetic resonance – 2020 update: Society for Cardiovascular Magnetic Resonance (SCMR): Board of Trustees Task Force on Standardized Post-Processing. J Cardiovasc Magn Reson 22:19. 10.1186/s12968-020-00610-6 PMC7066763 32160925

[22] Plein S, Schulz-Menger J, Almeida A et al (2011) Training and accreditation in cardiovascular magnetic resonance in Europe: a position statement of the working group on cardiovascular magnetic resonance of the European Society of Cardiology. Eur Heart J 32:793–798. 10.1093/eurheartj/ehq474 21289043

[23] OpenAI (2023) GPT-4 Technical Report. arXiv:2303.08774. https://arxiv.org/abs/2303.08774

[24] Koo TK, Li MY (2016) A Guideline of Selecting and Reporting Intraclass Correlation Coefficients for Reliability Research. J Chiropr Med 15:155–163. 10.1016/j.jcm.2016.02.012 PMC4913118 27330520

[25] Fleiss JL (1971) Measuring nominal scale agreement among many raters. Psychol Bull. 10.1037/h0031619

[26] Kaya K, Gietzen C, Hahnfeldt R et al (2024) Generative Pre-trained Transformer 4 analysis of cardiovascular magnetic resonance reports in suspected myocarditis: A multicenter study. J Cardiovasc Magn Reson 26:101068. 10.1016/j.jocmr.2024.101068 PMC11414660 39079602

[27] Hasani AM, Singh S, Zahergivar A et al (2024) Evaluating the performance of Generative Pre-trained Transformer-4 (GPT-4) in standardizing radiology reports. Eur Radiol 34:3566–3574. 10.1007/s00330-023-10384-x 37938381

[28] Salam B, Kravchenko D, Nowak S et al (2024) Generative Pre-trained Transformer 4 makes cardiovascular magnetic resonance reports easy to understand. J Cardiovasc Magn Reson 26:101035. 10.1016/j.jocmr.2024.101035 PMC10981113 38460841

