# Supplementary material for: Etiologic classification of suspected MINOCA using cardiovascular magnetic resonance reports: a comparison of a large language model and human readers
Source: Int J Cardiovasc Imaging. 2026 Mar 23;42(7):1395–406. doi: 10.1007/s10554-026-03689-7 (PMC13658433; doi:10.1007/s10554-026-03689-7)
Supplement: Supplementary file 1 [file 10554_2026_3689_MOESM1_ESM.docx]

**Supplementary Material 1**

**Prompt used for LLM-based diagnostic classification**

The following prompt was used for all cases without modification:

Dear ChatGPT,

We are conducting a research study investigating the performance of a large language model in the diagnosis of myocardial infarction with non-obstructive coronary arteries (MINOCA) based on cardiovascular magnetic resonance (CMR) findings.

Please read the following CMR report (written in German). Based solely on the information contained in the report, assign the case to one of the following eight diagnostic categories:

1. (Peri-)myocarditis
2. (Myo-)pericarditis
3. Takotsubo syndrome
4. Acute or subacute myocardial infarction
5. Ischemic myocardial injury due to coronary embolism, thrombosis, or dissection
6. MINOCA without an explanatory CMR finding
7. Cardiomyopathy (hypertrophic, dilated, or arrhythmogenic)
8. Systemic or infiltrative disease

Please provide the most likely diagnosis from the list above.
